# Current Status of Research on Osteoporosis after Solid Organ Transplantation: Pathogenesis and Management

**DOI:** 10.1155/2015/413169

**Published:** 2015-11-15

**Authors:** Gong-bin Lan, Xu-biao Xie, Long-kai Peng, Lei Liu, Lei Song, He-long Dai

**Affiliations:** Center of Organ Transplantation, The Second Xiangya Hospital of Central South University, Changsha 410011, China

## Abstract

Improved survival following organ transplantation has brought to the forefront some long-term complications, among which osteoporosis and associated fractures are the major ones that adversely affect the quality of life in recipients. The pathogenesis of osteoporosis in transplant recipients is complex and multifactorial which may be related to increased bone resorption, decreased bone formation, or both. Studies have shown that the preexisting underlying metabolic bone disorders and the use of immunosuppressive agents are the major risk factors for osteoporosis and fractures after organ transplantation. And rapid bone loss usually occurs in the first 6–12 months with a significant increase in fracture risk. This paper will provide an updated review on the possible pathogenesis of posttransplant osteoporosis and fractures, the natural history, and the current prevention and treatment strategies concerning different types of organ transplantation.

## 1. Introduction

Solid organ transplantation (SOT) has become an effective and established clinical therapy for end-stage renal, liver, cardiac, and pulmonary disease over the past two decades. Meanwhile, patient and graft survivals have been greatly improved due to tremendous advances in immunosuppressive agents and surgical techniques. However, improved survival has also made the adverse effects of some major complications apparent, such as osteoporosis and osteoporotic fractures.

The most rapid decrease in bone mineral density (BMD) frequently occurs in the first 6–12 months after transplantation and is accompanied with a marked increase in fracture risk. A recent study found that, compared with the non-SOT patients, the overall hazard ratio (HR) of osteoporosis after SOT was 5.14 (95% CI, 3.13–8.43), and the HR of related fractures was 5.76 (95% CI, 3.80–8.74) [1]. The highest risk of osteoporosis and fractures was observed in lung transplant recipients, followed by other types of SOT [1].

Studies have shown that pretransplant bone disease and posttransplant immunosuppressant therapy are the two major risk factors for osteoporosis. In addition, other factors including aging, tobacco, alcohol, nutritional deficiencies, immobility, and hypogonadism may also contribute to the risk of osteoporosis.

In this review, we will summarize the prevalence of osteoporosis and fractures and the current understanding on its etiology and natural history and will focus on the latest prevention and treatment strategies for osteoporosis and fractures in SOT recipients.

## 2. The Effects on Skeleton of Immunosuppressive Agents

### 2.1. Glucocorticoids

The therapeutic use of glucocorticoids (GCs) in patients has been shown related to an increase in bone loss and fracture risk [2]. Trabecular bone is usually more affected than cortical sites in glucocorticoid-induced osteoporosis (GIO) [3]. Patients who underwent organ transplantation are usually treated with high-dose glucocorticoid in the first few months and then taped gradually. However studies found that substantial bone loss occurring in early posttransplantation period has been largely attributed to treatment with high-dose glucocorticoid immediately after transplantation [4]. Daily doses of 7.5 mg prednisone equivalent are generally considered a major cause for bone loss, even though lower doses have been reported as harmful to bone health [5, 6].

Glucocorticoids in pharmacological dosages have an adverse effect on bone metabolism via both direct and indirect mechanisms [7, 8]. The direct effects of glucocorticoids on bone metabolism include influences on osteoblasts, osteoclasts, and osteocytes [9]. Studies reported that glucocorticoids may change the ratio between receptor activator of NF-*κ*B ligand (RANKL) and osteoprotegerin (OPG) in stromal and osteoblastic cells, which results in the induction of osteoclastogenesis and increased bone resorption [10, 11]. There is also evidence that GCs directly upregulate the expression of CSF-1, an essential cytokine for the survival of osteoclast precursors, leading to GC-induced born resorption [12].

The principal pathophysiological mechanism of GIO is decreased bone formation, owing to suppression of osteoblast differentiation and function, and promotion of osteoblast apoptosis [13]. Several molecular pathways underlying GC-induced reduction in differentiation potential have been identified, such as Wnt/*β*-catenin and bone morphogenetic protein 2 (BMP-2) signaling [14, 15]. The therapeutic GCs could suppress both Wnt/beta-catenin and BMP-2 signaling, which leads to a significant reduction in osteoblastogenesis and consequently to a loss of osteoblast-generated proteins [16]. GCs have also been reported to induce osteoblast apoptosis via an increase in proapoptotic factors of the Bcl-2 family such as Bim [17] and a reduction in *β*1-integrin expression [18], which eventually leads to bone loss and an increase in fracture risk.

Glucocorticoids could also indirectly affect bone and mineral homeostasis through various ways involving decreasing calcium absorption from the intestine, increasing renal excretion of calcium [19], reducing growth hormone secretion [20], and decreasing gonadal secretion of estrogen and androgens [21, 22], which may lead to osteoporosis and fractures. However, the main mechanisms underlying GIO are attributed to the direct influence of GCs on bone cells [23].

### 2.2. Calcineurin Inhibitors: Cyclosporine and Tacrolimus

The introduction of cyclosporine A (CsA) to immunosuppressive therapy in the early 1980s has significantly improved patient and allograft survival after organ transplantation [24]. CsA inhibits calcineurin and reduces T-cell function via suppression of regulatory genes expressing products such as interleukin-2, interleukin receptors, and the proto-oncogenes H-ras and c-myc [25]. The risk of fractures increases significantly in many patients treated with CsA because of rapid bone loss after transplantation [26–28]. Previous studies have proved that the effect of CsA on bone mineral metabolism is dependent on both the dose and the duration of administration [29, 30]. Studies in rats have indicated that CsA therapy at 7.5 mg/kg can damage the mechanical properties and thus may increase the fracture risk of lumbar spine [31]. Some* in vivo* study suggested that CsA may contribute to high-turnover bone loss, leading to an uncoupling of the dynamic bone remodeling cycle with resorption exceeding formation [32]. However, other scholars reported a lack of bone loss in 13 renal transplant patients receiving cyclosporine in a steroid-free regimen [33]. More interestingly, CsA monotherapy in renal transplant patients can significantly increase lumbar spine BMD while recipients treated with both CsA and glucocorticoids present a significant decrease [34]. Up to now, the precise mechanisms involved in CsA induced bone loss are still not well defined.

Tacrolimus (FK506), another calcineurin inhibitor, inhibits T-cell activation and proliferation and cytokine gene expression and also causes trabecular bone loss in rats [25]. The possible mechanism of FK506 on bone metabolism is mainly due to an excess of bone resorption over formation involving the imbalance of RANK/RANKL/OPG system [35] or partly by inhibiting ERK1/2 pathway [36]. Both liver [37] and cardiac [38] transplant recipients have been shown to sustain rapid bone loss with tacrolimus-based immunosuppression. However, it has been found that lesser reduction in bone mineral density occurs with FK506, compared with CsA in rats [39]. Similarly, liver transplant treatment with FK506 showed a more favorable long-term effect on bone mass evolution than CsA therapy, probably due to the lower dose of glucocorticoids used in the FK506 [40]. A recent study of renal transplant patients found that those with high FK506 blood concentrations were at greater risk of bone loss [41].

### 2.3. Mammalian Target of Rapamycin Inhibitors (mTORi): Sirolimus and Everolimus

Sirolimus (SIR) and everolimus (EVE) are anti-mTOR drugs commonly used in transplant recipients. It is well known that mTORi present both antiproliferative and antiangiogenic activities; thus their interference with bone metabolism is unavoidable. Alvarez-Garcia et al. found that SIR slowed down growth and caused marked alterations in the growth plate of young rats [42]. In a cross-section study of renal transplant recipients, SIR-based regimen was associated with lower serum markers of osteoclast activity and maturation in contrast to a CI-based immunosuppression [35], which was in agreement with the experiment data from other researchers [43]. In a previous animal study, SIR was reported to be a bone sparing immunosuppressant compared with tacrolimus and cyclosporine after administration for 28 days in rats [44]. mTOR/S6 K has been identified as an essential signaling pathway engaged in the stimulation of cell survival in osteoclasts [45]. Kneissel et al. studied the impact of EVE on mouse and human osteoclasts* in vitro* and on bone in an ovariectomized rat model and found EVE can suppress cancellous bone loss, bone resorption, and cathepsin K expression by osteoclasts [46]. Westenfeld et al. also believed that EVE may exert beneficial bone effects, potentially reducing bone resorption and contributing to a bone-protective effect [35]. 

### 2.4. Other Immunosuppressive Agents

Immunosuppressive drugs such as MMF and azathioprine had no deleterious effect on bone in rat model [47, 48]. There is little information available regarding the skeleton effects of other immunosuppressant agents.

Table 1 shows the effects of immunosuppressive drugs on skeleton.

## 3. Different Types of Transplantation

### 3.1. Renal Transplantation

Patients with end-stage renal disease (ESRD) are usually suffering from renal osteodystrophy [49]. It has been reported that renal osteodystrophy persists after transplantation because of elevated levels of PTH and fibroblast growth factor 23 (FGF-23), though at lower levels compared to pretransplantation [50]. Several factors have been demonstrated to be associated with osteopenia after renal transplantation including preexisting bone diseases, secondary hyperparathyroidism, and low bone mineral density due to advanced age and/or vitamin D deficiency, as well as posttransplant immunosuppressive therapy, steroid dose, and ongoing disorder in the phosphate-calcium-parathyroid hormone-vitamin D axis [51, 52].

The loss of bone mass is particularly prominent during the first 6 months after renal transplantation and bone mineral density (BMD) decreases to a mean of 5.5%–19.5% [53] and 2.6%–8.2% between the 6th and 12th month [54]. After 1 year, it is only 0.4%–4.5% of the mean [54]. The prevalence of “osteopenia” and “osteoporosis” after renal transplant has been reported to range from 25% to 35% and 8% to 28%, respectively [55–57]. A cross-sectional analysis of 389 stable renal transplant recipients has showed that osteopenia or osteoporosis is more prevalent at the femoral neck than lumbar spine [58]. The main pathology of bone remodeling following transplantation is considered to include decreased bone formation and mineralization associated with persistent bone resorption [59].

The incidence rate of fractures has been reported to vary between 7% and 11% in nondiabetic renal transplant recipients but is much higher in patients with diabetic nephropathy [60]. The recipients experienced a 3.6–3.8-fold higher risk of fracture over general population, and it is 30% higher in the first 3 years after transplantation than patients undergoing dialysis [60]. And the recipients fractured mainly in appendicular sites (hips, long bones, ankles, and feet) rather than axial sites (lumbar spine and ribs) [61]. Sukumaran Nair et al. reported that the hip fracture incidence was almost 5 events per 1000 person-years in the first posttransplant year, then closer to 3 events per 1000 person-years in the 2 subsequent years [62]. Patel et al. studied 165 renal transplant patients and found that 27 patients had either vertebral deformities or a history of a low trauma fracture after transplantation [63]. A systematic review of 262,678 recipients showed that the 5-year cumulative incidence of fracture varied ranging from 0.85% to 27% [64]. Recently, Sukumaran Nair et al. reported that case-mix adjusted hip fracture rates after renal transplantation declined substantially since 1997, partly benefitting from the improvements in immunosuppressive therapy [62]. 

A longitudinal study displayed that persistently high PTH level (>130 ng/L) at 3 months after transplantation was a major independent risk factor for fracture [65]. Nikkel et al. identified some other factors that were also associated with increased fracture risk, such as older age, white race, prior dialysis, and pretransplant fracture [66].

### 3.2. Cardiac Transplantation

Osteoporosis is very common among patients with end-stage heart disease ranging between 8% and 23% [67, 68]. Longitudinal studies have found that BMD may decrease by 10% to 20% before cardiac transplantation [69]. Rapid bone loss occurs most often in the first year after transplantation. Lumbar spinal BMD declines by 3% to 10% and femoral neck BMD by 6% to 11% during the first year [70], which is considerable when compared with the 1.41% decrease at the lumbar spine and 0.35% at the femoral neck in healthy population [71]. BMD begins to stabilize in the second year and may even increase after the third year [70]. Bone loss occurring shortly after heart transplantation is probably due to immunosuppressive therapy with high-dose glucocorticoids and calcineurin inhibitors, particularly cyclosporine [72]. Moreover, vitamin D deficiency and testosterone deficiency (in men) have also been considered to be associated with more severe bone loss [73].

The prevalence of fragility fractures is estimated between 22% and 44% among cardiac transplant recipients, and the incidence of fractures during the first three years after transplantation ranges from 18% to 35% [74, 75]. The relationship between BMD and fractures was reported as weak, because lots of patients underwent a fracture at a relatively normal BMD [76]. In some study, a low incidence of fracture was found even though bone loss was evident [77].

In a cross-sectional study of 157 patients within less than 10 years from cardiac transplantation, the authors found fractures by spine X-ray in 40% of subjects, whereas osteoporosis by DXA was present only in 13% of lumbar spine and 25% of hip scans. While *T*-score threshold was modified to −1.5 standard deviations, the prevalence of fractured increased significantly, reaching 60%. Thus they thought that the standard densitometric criteria were unreliable to identify bone fragility after cardiac transplantation, and a different threshold should be considered [78].

In a longitudinal study by Wang et al., multivariate analysis showed that pretransplant BMD at either site and/or a history of fracture were significant predictors for developing osteopenia or osteoporosis after cardiac transplantation [67]. Other authors found, besides lumbar spine BMD before transplantation, age was also an important predictor of fracture [74].

### 3.3. Liver Transplantation

Bone disorders in patients with chronic liver disease are termed as hepatic osteodystrophy, which includes osteoporosis and, less frequently, osteomalacia. The prevalence of bone disease has been reported to range from 12% to 55% among patients with cirrhosis [79–82]. In a study by Bai et al., the risk of low BMD was significantly correlated with the primary liver disease and elevation of serum iPTH level when using univariate analysis [83]. Bone disorders after liver transplantation can be related to many risk factors, for example, preexisting hepatic osteodystrophy, malnutrition, immobility, hypogonadism, and immunosuppressant.

Following liver transplantation, a rapid decline in BMD mainly occurs in the first 3 to 6 months [84, 85] and is associated with a significant increase in the incidence of osteoporotic fractures. One bone histology study has demonstrated that bone loss halts approximately 6 months after liver transplantation and tends to increase, especially in lumbar spine, which leads to the recovery of bone mass within 2 years after the surgery [86]. The raise in BMD is significantly higher among premenopausal than perimenopausal and postmenopausal women, probably owing to the protective estrogen effect on the skeleton [87].

The fracture incidence after successful liver transplantation is relatively high and ranges between 10% and 43% [74, 88]. The majority of fractures take place during the first 2 years, mostly in lumbar spine [79]. Preexistent low BMD before transplantation has been regarded as the major risk factor for posttransplantation fractures [83]. Besides, some conditions such as pretransplant fractures as well as older age, the type of liver disease, and retransplantation also contribute to the development of fractures [89].

### 3.4. Lung Transplantation

Osteoporosis is prevalent in patients who are candidates for lung transplantation, especially among those with chronic obstructive pulmonary disease (COPD). The prevalence of osteoporosis and osteopenia varied as 9–69% and 27–67% in COPD patients, respectively [90]. Contributing factors include older age, female gender, low BMI, history of fractures, and tobacco exposure [91]. Steroid consumption has been regarded as a major risk factor [91, 92]. Furthermore, raised high density lipoprotein cholesterol levels are also considered to be independently associated with osteoporosis [93].

Rates of bone loss at lumbar spine and femoral neck vary between 2% and 5% in the first year after lung transplantation, respectively [67]. Pretransplant BMD is an important predictor of subsequent osteopenia or osteoporosis after transplantation [67].

There are limited studies evaluating fracture risk following lung transplantation. In an earlier study, symptomatic fracture rated up to 15–50% and a loss of BMD of 4% to 12% occurred in the first year after lung transplantation [94]. In a very recent research, Hariman et al. retrospectively analyzed 210 medical records of patients receiving lung transplantation and found 17 patients (8.0%) had fractures after transplantation. The incidence of fracture in lung transplant patients in years 1 to 5 was 4.8%, 1.0%, 0.0%, 1.0%, and 1.9%, respectively [95], probably owning to the early initiation of antiresorptive therapy. Another study conducted by Cahill et al. showed that antiresorptive therapy with pamidronate preserved or increased BMD prior to and following transplantation, resulting in only 4% of new fractures [96].

## 4. Management of Bone Disease before and after Organ Transplantation

### 4.1. Pretransplantation Assessment

Since bone disease is very common in patients with end-stage organ disease, all patients awaiting transplantation should be given comprehensive evaluation. Bone densitometry of the lumbar spine and hip measured by DXA should be performed routinely. Spine radiographs should be measured to diagnose prevalent fractures. Any secondary causes of osteoporosis should be identified and treated. Some conditions such as hypogonadism, vitamin D deficiency, and secondary hyperparathyroidism must be corrected whenever possible. In addition, candidates for transplant should be encouraged to change detrimental lifestyle such as immobilization, smoking, and alcohol abuse. Furthermore, all patients should be administered the recommended daily allowance for calcium (1000–1500 mg/d) and vitamin D (400–800 IU/d).

### 4.2. Posttransplantation Management

It is well known that both pretransplant bone disorders and immunosuppressive agents lead to rapid bone loss and increased fracture risk following transplantation. Therefore, prevention therapy should be adopted immediately after transplantation. Perhaps the low-dose use of GCs in a shortest period is of the greatest importance. Besides, at present, there are several kinds of drugs available for prevention and treatment of posttransplant osteoporosis.

### 4.3. Active Vitamin D Metabolites

Vitamin D deficiency is prevalent in pre- and posttransplant patients. In a cross-sectional study of 173 renal transplant recipients, moderate-to-severe vitamin D deficiency was observed in 29% of the patients, with a further 51% of insufficiency [97]. The active vitamin D metabolites have been proved effective on the prevention and treatment of osteoporosis. The probable underlying mechanisms include that they may overcome GC-induced decreases in intestinal calcium absorption, reduce the potential for secondary hyperparathyroidism, promote the differentiation and maturation of osteoblast precursors, and potentiate the immunosuppressive action of CsA [98].

Torres et al. showed in a double-blind, randomized and controlled trial that renal transplant recipients who received intermittent calcitriol (0.5 *μ*g/48 h) for 3 months, plus oral calcium supplementation (0.5 g/day) during 1 year, had less bone loss than those with calcium alone [99]. Similarly, other authors observed that treatment with active vitamin D (0.25 *μ*g/d) and calcium could prevent bone loss at the lumbar spine and proximal femur during the first 6 months after renal transplantation [100].

However, there are some contradictory results. Another double-randomized study of heart or lung transplant recipients who were given placebo or calcitriol (0.5–0.75 *μ*g/d) for either 1 or 2 years after transplantation found that bone loss at the proximal femur was significantly reduced compared with placebo group, but there was no significant difference in lumbar spine BMD between groups [101].

### 4.4. Bisphosphonates

Bisphosphonates are currently the most commonly used medications for the treatment of osteoporosis. They inhibit osteoclast-mediated bone resorption through several mechanisms as yet not fully elucidated. Oral or intravenous bisphosphonates have been regarded as the most promising approach for the management of posttransplant osteoporosis [4]. Compared head-to-head with vitamin D sterols, bisphosphonates had greater efficacy on improving bone mineral density after transplantation [102].

In a recent meta-analysis regarding the value of pamidronate to prevent bone loss of lumbar spine and femoral neck in the first year of renal transplantation, 6 randomized trials including 281 patients were identified. Among all 6 trials, administration of pamidronate was associated with significant reduction of bone loss in the lumbar spine compared with the control group. No major side effects were reported [103]. Similar results have been observed in a randomized controlled trial of patients with liver transplantation [104, 105] and in studies of cardiac [105] and lung [96] transplant recipients independent of the time following transplantation.

A 1-year randomized, double-blind, placebo-controlled study of 129 renal transplant recipients with early stable renal function (≤28 days after transplantation, GFR ≥ 30 mL/min) was conducted by Smerud and his colleagues. They found that patients with intravenous 3 mg ibandronate every 3 months for 1 year as add-on to calcitriol and calcium had a significant improvement of BMD in total femur and ultradistal radius [106]. In a randomized study of male cardiac transplant patients, researchers found that intravenous ibandronate not only preserved bone mass, but also reduced vertebral fracture risk and prevented uncoupling of bone formation and resorption [107], which was in accordance with another study of liver transplant recipients [108].

Another intravenous bisphosphonate, zoledronic acid, has also been used in a randomized placebo-controlled trial of 20 patients after renal transplantation. It was administrated in a dose of 4 mg twice within 3 months after surgery. At the end of 6 months, it was shown that zoledronic acid improved the calcium content of cancellous bone and increased lumbar spine BMD [109].

In a case-control study, short-term weekly use of alendronate (Fosamax), an oral bisphosphonate, could significantly improve both lumbar spine BMD and bone condition, regardless of effect of immunosuppressant after a long period after renal transplantation [110]. In a long-term of low-dose weekly use of alendronate (35 mg/week) for long-term renal transplant recipients, authors found alendronate could suppress bone turnover but not fracture in kidney transplant recipients with hyperparathyroidism and chronic kidney disease [111].

### 4.5. Calcitonin

Studies of calcitonin therapy in transplant patients with osteoporosis are scarce. Preliminary data from a single-center cross-sectional study has indicated that calcitonin may help to restore bone mass in kidney transplant recipients with osteoporosis [112].

### 4.6. Antibody to the Receptor Activator of Nuclear Factor-kB Ligand

Denosumab is a fully human monoclonal antibody to the receptor activator of nuclear factor-kB (RANK) ligand that blocks binding to RANK, inhibiting bone resorption and increasing bone density. In a large clinical trial involving 7868 postmenopausal women aged 60 and 90, administration of denosumab twice yearly for 3 years was associated with a significant reduction in the risk of vertebral, nonvertebral, and hip fractures in women, as compared with placebo [113]. In a dose-response-based meta-analysis including 142 randomized controlled trials, 3 years with denosumab resulted in bigger changes in BMD of lumber spine and total hip compared with 3 years of treatment with 10 mg/d oral alendronate, 5 mg/y i.v. zoledronic acid, 5 mg/d oral risedronate, 150 mg/mo oral ibandronate, 3 mg i.v. ibandronate every 3 months, 60 mg/d oral raloxifene, and 200 IU/d calcitonin [114]. Given its unique actions, denosumab may be useful for the prevention and treatment of osteoporosis and fractures in organ transplant patients.

### 4.7. Teriparatide

Teriparatide is a recombinant human parathormone (PTH 1–34) currently used among patients with osteoporosis who are at high risk for fracture [115]. Only limited data are available on teriparatide therapy for patients receiving organ transplantation. In a randomized double-blind trial of 26 kidney transplant patients, daily subcutaneous injection teriparatide (20 *μ*g) of 6 months was well tolerated but did not improve either BMD or bone turnover early after transplantation [116]. However, for the kidney transplant patients with severe hypocalcemia and low PTH, treatment with teriparatide showed good results without side effects [117].

### 4.8. Hormone Replacement Therapy (HRT)

Hormonal replacement therapy has been proposed to reduce the risk of osteoporosis in menopausal women. However, there is restricted literature regarding effect of HRT on transplant recipients. One study was reported by Isoniemi et al. in 33 postmenopausal female liver transplant recipients with 2-year follow-up [118]. The lumber spine BMD increased by 5.3 and 1.2%, respectively, in the first and second year, while the femoral neck BMD increased by 3.3 and 1.2%. Only 20% of the patients had osteoporosis after 2 years of treatment, whereas over 50% of the women had osteoporosis at baseline. Another 10-year-experience study showed, with the treatment of estradiol and progestin, total regression of climacteric symptoms was reported in 75% of kidney transplant patients without negative effect [119]. Further studies are needed to establish the efficacy and safety of HRT among transplant recipients.

## 5. Conclusion

In recent years, more and more attention has been drawn to new therapeutic challenges in the long-term management of SOT recipients due to the greatly improved patient and graft survival. And posttransplant osteoporosis is one of them. Although the pathogenesis is not well established, preexisting bone disorders and transplant-specific therapies appear to play a key role. At present, there is no golden standard for the prevention and treatment of osteoporosis and fractures following organ transplantation. However, treatment with vitamin D and/or bisphosphonate in the first year following transplantation was associated with a reduction in the incidence of osteoporosis and fractures. Other medications including calcitonin, teriparatide, and HRT have also been evaluated in some cases. More research is needed to verify the effect of various therapeutic approaches and develop a clinical guideline of therapy strategy for bone disorders after organ transplantation.

## Figures and Tables

**Table 1:** Effects of immunosuppressive agents on skeleton.

Immunosuppressive drugs	Effects on skeleton
Glucocorticoids	
Direct effects	Inhibit bone formation
	Stimulate bone resorption
Indirect effect on skeleton	Reduce intestinal calcium absorption
	Increase urinary calcium excretion
	Decrease growth hormone secretion
	Decrease gonadal secretion of estrogen and androgens

Calcineurin inhibitors^*∗*^	
Cyclosporine A and tacrolimus	High-turnover osteopenia with resorption exceeding formation
Sirolimus and everolimus^*∗*^	Inhibit bone resorption
Mycophenolate mofetil^*∗*^	No effect on bone volume
Azathioprine^*∗*^	No effect on bone volume
Mizoribine	Unknown

^*∗*^These observations are based primarily on animal studies.
